# Evolution of Red Algal Plastid Genomes: Ancient Architectures, Introns, Horizontal Gene Transfer, and Taxonomic Utility of Plastid Markers

**DOI:** 10.1371/journal.pone.0059001

**Published:** 2013-03-25

**Authors:** Jan Janouškovec, Shao-Lun Liu, Patrick T. Martone, Wilfrid Carré, Catherine Leblanc, Jonas Collén, Patrick J. Keeling

**Affiliations:** 1 Department of Botany, University of British Columbia, Vancouver, British Columbia, Canada; 2 Department of Life Science, Tunghai University, Taichung, Taiwan; 3 UPMC-University of Paris VI, Station Biologique, Roscoff, France; 4 CNRS FR2424, ABiMS, Station Biologique, Roscoff, France; 5 CNRS, UMR 7139 Marine Plants and Biomolecules, Station Biologique, Roscoff, France; Rutgers University, United States of America

## Abstract

Red algae have the most gene-rich plastid genomes known, but despite their evolutionary importance these genomes remain poorly sampled. Here we characterize three complete and one partial plastid genome from a diverse range of florideophytes. By unifying annotations across all available red algal plastid genomes we show they all share a highly compact and slowly-evolving architecture and uniquely rich gene complements. Both chromosome structure and gene content have changed very little during red algal diversification, and suggest that plastid-to nucleus gene transfers have been rare. Despite their ancient character, however, the red algal plastids also contain several unprecedented features, including a group II intron in a tRNA-Met gene that encodes the first example of red algal plastid intron maturase – a feature uniquely shared among florideophytes. We also identify a rare case of a horizontally-acquired proteobacterial operon, and propose this operon may have been recruited for plastid function and potentially replaced a nucleus-encoded plastid-targeted paralogue. Plastid genome phylogenies yield a fully resolved tree and suggest that plastid DNA is a useful tool for resolving red algal relationships. Lastly, we estimate the evolutionary rates among more than 200 plastid genes, and assess their usefulness for species and subspecies taxonomy by comparison to well-established barcoding markers such as *cox1* and *rbcL.* Overall, these data demonstrates that red algal plastid genomes are easily obtainable using high-throughput sequencing of total genomic DNA, interesting from evolutionary perspectives, and promising in resolving red algal relationships at evolutionarily-deep and species/subspecies levels.

## Introduction

Red algae are an ancient lineage of ecological importance that play a prominent role in our thinking about the evolutionary history of photosynthesis in eukaryotes. Despite their importance, however, genomic data from red algae remain scarce. To date, only one complete nuclear genome and less than a dozen organellar genomes have been characterized and described. Red algal plastid genomes are particularly interesting, because they contain the largest gene complement among all known plastids, are compactly organized, and comparatively slowly evolving. This suggests that among modern plastid DNAs they might best represent the ancestral state of primary plastids also present in land plants, green algae and glaucophytes. However, only five complete plastid genome sequences from red algae have been described: two from the early-branching extremophiles (*Cyanidium caldarium* [1] and *Cyanidioschyzon merolae* [2]) and three from the multicellular group comprising Bangiales (*Porphyra purpurea* [3], *P. umbilicalis* [4]) and florideophytes (*Gracilaria tenuistipitata* var. *liui* [5]). The florideophytes are particularly diversified, and ecologically and industrially important subgroup of red algae, so the lack of genome data from this lineage is significant. Several next-generation sequence datasets have recently become available, but plastid DNA or transcripts were not characterized in the data prior to this study (e.g., *Grateloupia lanceola* [6], and *Porphyridium cruentum* [7]. At the same time, a comprehensive comparison of red algal plastid genomes has also been absent or biased by non-unified annotations. A good example of this is an unpublished plastid sequence from *Pyropia yezoensis* (Bangiales, renamed according to [8]), which has long been available, but never compared to the other taxa, prohibiting any useful analysis of shared versus unique features across the group. Similarly, plastid introns from red algae have long been known [9], but generally remained uncharacterized, or perhaps even overlooked (such as in *Gracilaria tenuistipitata* var. *liui*, see below). Overall, better sampling of red algal plastid genomes and a more detailed comparison between them are needed to fully appreciate the implications of their remarkable characteristics and how these characteristics relate to other photosynthetic eukaryotes, particularly to those with secondary red algal plastids [10].

Red algal plastid genes have also shown promise as markers for reconstructing evolutionary relationships [11], [12] and species barcoding [13]. So far, however, only a fraction of plastid genes (10 out of more than 200) have been tested for their phylogenetic utility [11], [12], and the phylogenetic potential of plastid genomes as a whole remained unexplored. High-throughput sequencing methods make plastid genomes even more relevant, because complete plastid genomes could be easily assembled from total genomic DNA sequences, generating comparable sets of phylogenetic markers. Similarly, plastid genes are an obvious source of markers for barcoding and species delimitation, because they can be easily amplified using previously described PCR assays. Mitochondrial cytochrome oxidase (*cox1*) and plastid rubisco (*rbcL*) have become the most commonly used gene for red algal barcoding [13], [14]. However, the resolution of these markers may not be optimal in some lineages and at the population level in particular, with *rbcL* already providing lower resolution in at least some cases [15], [16]. A comprehensive comparison of new markers and their potential ability to discriminate species, subspecies, and populations is important, but few have been evaluated [17], [18] and a genome-wide assessment of evolutionary rates to identify such markers has been restricted by limited genome sampling.

Here, we expand the available plastid pool from red algal plastid data by characterizing three new complete and one partial plastid genome. We show that they all have compacted and conserved architectures that are slowly-evolving. They also contain several unique features, such as a group II intron in the plastid *trnMe* gene, and a rare case of horizontally acquired *leuC/D* operon. We demonstrate that whole-plastid genomes are a promising resource to resolve red algal relationships, and that they contain a range of markers potentially useful for species barcoding.

## Results and Discussion

**Figure 1:**
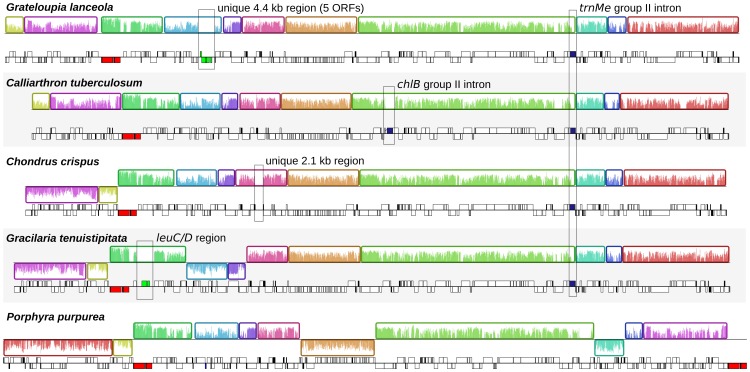
Overview of red algal plastid genomes. Linearized maps of complete florideophyte plastid genomes compared with the bangialean *Porphyra*. For each genome, colour-coded syntenic blocks are shown above and gene maps are shown below. Syntenic blocks above the horizontal line are in the same strand while those below the line are on the opposite strand. Horizontal bars inside the syntenic blocks show sequence conservation. Block boundaries correspond to sites where inversion events occurred. In gene maps, genes above the horizontal line are transcribed left to right while those below are transcribed from right to left. Unique regions are boxed with genes in green, regions with introns are boxed with intronic segments in blue, and rRNA operons are red.

The complete plastid genomes of *Calliarthron tuberculosum*, *Chondrus crispus,* and *Grateloupia lanceola*, and the partial genome of an unspecified *Cruoria* species (as determined by sequences in this study; Materials and Methods) were assembled from 454-pyrosequencing and Solexa reads, and manually annotated. All previously available plastid genomes from red algae were re-annotated taking advantage of these new genomes for comparison (*P. umbilicalis*, a close relative of *P. purpurea* was excluded). Altogether, this resulted in 118 corrections to existing annotations affecting the presence of genes and introns, start codon positions, open reading frame (ORF) prediction, and frameshifts (Table S1; Materials and Methods). With these updated and unified annotations, we re-analysed annotation-sensitive features (i.e., intergenic lengths, coding density, etc.), and other general characteristics across all red algal plastid genomes (Table 1). All the newly sequenced plastid genomes map as circles and consistently fall among the largest plastid genomes sequenced to date, ranging from 179 to 188 kbp. Together with other red algae, these genomes contain the most extensive gene complements of all plastids (238 to 250 genes in florideophytes and Bangiales), including dozens of cyanobacterial genes absent in other plastids (Table 1, Figure 1). In a noteworthy contrast to the retention of cyanobacterial genes, many red algal plastid genomes do not contain species-specific ORFs, which are numerous in plastids from other lineages. Two exceptions to this rule appear to have different causes: firstly, four ORFs were found to be shared among multiple red algal species suggesting that they may encode for functional products (Table 1). Secondly, five ORFs (comprising a unique 4.4 kb region) were found to be inserted in an otherwise conserved region in *Grateloupia*, indicative of a secondary origin possibly from outside of the plastid genome (Table 1). All florideophytes have retained a single copy of the plastid ribosomal RNA (rRNA) operon suggesting this state was also present in their common ancestor (Figure 1). This implies that the ancestral inverted rRNA repeat, which is still retained in most secondary red alga-derived plastids, has been rearranged into a linear repeat in the Bangiales, and one of its copies independently lost in florideophytes and Cyanidiales. Our sequencing approach demonstrates that red algal plastid genomes are easily obtainable using high-throughput shotgun sequencing and that many red algae have retained large and gene-rich genomes with various putatively ancestral features, but also some unexpected and unique characteristics, described below.

**Table 1:** General characteristics of red algal plastid genomes^1^.

Species^2^	Ppu	Pye	Ctu	Ccr	Gte	Gla	Cca	Cme	NOTABLE FEATURES
Group	Bangiales		florideophytes				Cyanidiales		
Total length [bp] 3	191028	191954	178981	180086	183885	188384^4^	164923	149987	some of the largest plastid genomes
Total % GC^5^	33	33.1	29.2	28.7	29.2	30.3	32.7	37.6	
Unique genes	250	250	238	240	240	244	223	234	most gene-rich plastid genomes known
Protein-coding(% GC)	209 (33.7)	209 (33.9)	201 (30.0)	204 (29.8)	204 (30.4)	208 (31.6)	189 (33.3)	197 (37.3)	
uknown ORFs(sp-spec^6^)	3 (0)	3 (0)	2 (0)	3 (0)	3 (0)	8 (5)	1 (0)	2 (2)	species-specific ORFs largely absent
rRNA genes (% GC)	3 (49.3)	3 (49.4)	3 (47)	3 (47)	3 (46.2)	3 (47.9)	3 (45.4)	3 (52.5)	rRNA repeat lost in florideophytes and Cyanidiales
tRNA genes (% GC)	35 (53.8)	35 (53.0)	31 (51.2)	30 (50.9)	30 (50.7)	30 (52.6)	30 (50.6)	31 (53.5)	largest tRNA gene complement in Bangiales
small RNA genes	3	3	3	3	3	3	1	3	
Introns (all group II)	0	0	2	1	1	1	0	0	tRNA gene intron in florideophytes
Median intergenic size [bp]	77	79	61	68	76	85	56	10	massive intergenic reduction in *Cyanidioschyzon*
Mean intergenicsize [bp]	106.5	110	90.1	106.9	113.2	125.8	83.4	37.7	compacted organization

1 All values are based on updated annotations (Materials and Methods and Table S1).

2 Ppu = Porphyra purpurea str. Avonport (NC_000925), Pye = Pyropia yezoensis str. U-51(NC_007932), Ctu (Calliarthron tuberculosum; KC153978), Ccr (Chondrus crispus; HF562234), Gte (Calliarthron tuberculosum; KC153978), Ccr (Chondrus crispus; HF562234), Gte (Gracilaria tenuistipitata var. liui; NC_006137), Gla (Grateloupia lanceola; HM767098 and HM767138), Cca (Cyanidium caldarium str. RK1; NC_001840), Cme (Cyanidioschyzon merolae str. 10; NC_004799).

3 base pairs.

4 several bases in two stem-loop regions may be missing (see Materials and Methods).

5 GC content.

6 species-specific ORFs (not counting ORFs that are shared among multiple species).

### Compaction is Consistently High, but Species- not Lineage-specific

Red-algal plastid genomes are highly compact, but with the updated annotations we observed that the average and median intergenic lengths varied within lineages (see [2]). The median intergenic distance ranged from 61 to 85 base pairs (bp) in Bangiales and florideophytes, and decreased to only 10 bp in the deep-branching *Cyanidioschyzon merolae*. These estimates revealed that while compaction is consistently high, there is species specificity. At median 61 bp intergenic distance the *Calliarthron* plastid genome is more similar to that of *Cyanidium* (56 bp) than to other florideophytes. This in turn suggests that the extraordinary compaction of the plastid genome in *Cyanidioschyzon* has likely resulted from species-specific factors after the divergence of *Cyanidioschyzon* from other acidothermophilic cyanidiales, and does not relate to ancestral adaptation of cyanidiales to their acidothermophilic environments [19]. Similarly, no obvious correlation between the plastid genome compaction and phylogenic affinity was observed in florideophytes and Bangiales. Mean intergenic sizes were roughly proportional to medians, but generally higher, affected by several large intergenic outliers. These large spacers were primarily associated with the rRNA operon, the 5-ORF region in *Grateloupia* (see Figure 1), and the *Gracilaria leuC/D* region acquired by horizontal gene transfer (see bellow); the only outstanding case was a unique 2.1 kb spacer between *trnS* and *trnD* in *Chondrus* of an unknown origin.

### Highly Conserved Genome Architectures with an Interesting Exception in *Pyropia*

Comparing gene presence and order among the six complete sequences from the Bangiales and florideophytes showed that, despite their evolutionary distance, the genomes are highly co-linear (Figure 1). Three orthologous gene clusters account for all genes among all florideophytes, and only 11 orthologous gene clusters account for all genes among florideophytes and Bangiales, all of which could be readily reconstructed through a small number of inversion events (Figure 1). *Grateloupia* and *Calliarthron* plastid genomes were co-linear. *Chondrus* had the smallest re-arrangement distance to the other plastid genomes and differed by only three inversions from the bangialian *Porphyra,* suggesting that it may most closely reflect the ancestral gene organization in florideophytes. However, even the most divergent genome pair (*Gracilaria* and *Porphyra*) could be reconstructed by mere 5 rearrangements. Considering the substantial evolutionary distance among these red algae compared to other photosynthetic groups [20], it seems likely that their plastid genomes represent some of the most slowly-evolving genome architectures among all plastids, paralleled only by those of some streptophytes [21]. The plastid genomes of the two Bangiales, *Porphyra* and *Pyropia*, are co-linear, except for the orientation of five individual genes. Interestingly, all five of these gene inversions occur in otherwise highly conserved gene clusters suggesting they altered ancient polycistronic operons. Three of these inversions (*rps11*, *rps13*, and *rpl31*) are located in the ribosomal supercluster, an array of 29 genes that is conserved across the whole red plastid lineage (Figure S1, [22], except for rare rearrangements in the alveolates [10], [23]. Two more single gene inversions affected *cemA*, otherwise conserved in all red algae, and *petD* in the *petB/petD* cluster. The latter case is noteworthy, because *petB* and *petD* are transcribed polycistronically in land plants [24], [25] and are adjacent in majority of all plastids. Comparisons to other plastid genomes shows that all five inversions were recent events in the lineage leading to *Pyropia*, suggesting that there are lineage-specific changes to conservation of genome architecture as well: in this case *Pyropia* acquiring abundant single gene inversions when reorganization is generally slow.

### Gene Contents Suggest a Slow Rate of Plastid-to-nucleus Gene Transfer

**Figure 2:**
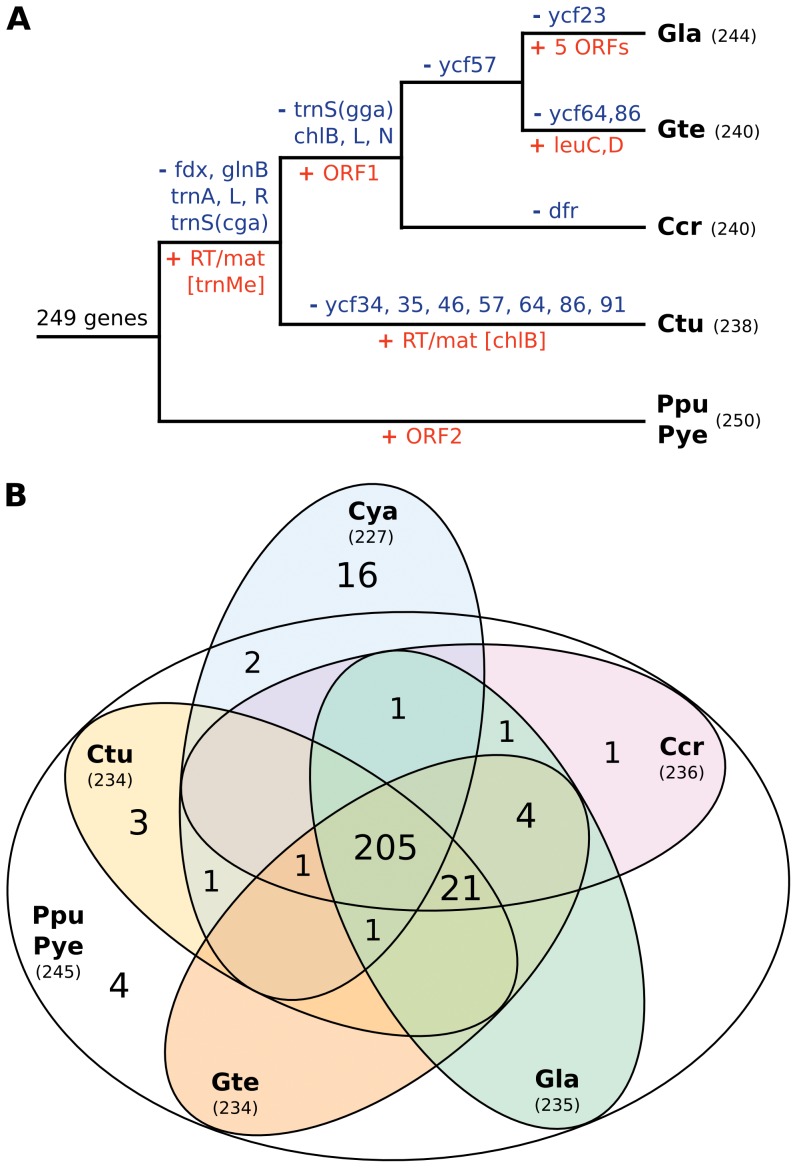
Gene content, gain and loss in red algal plastid genomes. (A) Gain and loss of genes in the florideophyte and Bangiales plastid genomes. Losses of cyanobacterial genes (blue), and gene gains (red) are mapped onto a schematic phylogeny (see Figure 5) using maximum parsimony. Note that losses may have occurred later (independently), whereas gains may have occurred earlier (loss in early-branching lineage) than inferred under this criterion. RT/mat abbreviates a reverse transcriptase/maturase ORF and the intron-bearing gene is in squared brackets. The absence of magnesium chelatase (chl), tRNA genes (trn), and unknown conserved genes (ycf) may represent outright losses rather than transfers to the nucleus in some cases. (B) Comparison of cyanobacterial gene content in red algal plastids including Cyanidiales. Venn diagram is showing number of cyanobacterial genes shared among red algal plastid genomes, which represented by bubbles. Ppu *Porphyra purpurea*, Pye *Pyropia yezoensis*, Ctu *Calliarthron tuberculosum*, Ccr *Chondrus crispus*, Gte *Gracilaria tenustipitata* var. *liui*, Gla *Grateloupia lanceola,* Cya Cyanidiales (sum of genes in *Cyanidium caldarium* and *Cyanidioschyzon merolae*).

An examination of gene content revealed that *Porphyra* and *Pyropia* have retained the largest gene complement among all plastids: 250 unique genes including 245 genes of unambiguous cyanobacterial descent. 249 of these genes were also present in their common ancestor with florideophytes (Figure 2A, B). This suggests that little or no gene loss has occurred in the plastids of Bangiales since their divergence from florideophytes, which is believed to have occurred more than 500 million years ago [20]. Since many of the same genes have been transferred to the nucleus in other algae, this points to a comparatively slow rate of gene transfer in the Bangiales that is unprecedented in any other plastid lineage. However, other red algal plastids are not far behind: the florideophytes still encode 95–96% of genes ancestrally shared with Bangiales. Out of the 19 differentially retained genes, six appear to have been lost from the plastid prior to the florideophyte divergence, and 13 lost more recently, with *Chondrus* retaining the greatest number of ancestral genes (8) and *Gracilaria* and *Calliarthron* retaining the least (6) (Figure 2A). However, eight of these events are likely due to the outright loss of the gene in question, and not due to transfer to the nucleus: these genes encode redundant tRNA variants and subunits of the plastid light-independent protochlorophyllide reductase, all of which have been completely lost in many other photosynthetic eukaryotes. In addition, most of the remaining ‘transfers’ involve conserved genes of unknown function (*ycf*s) and might therefore also represent outright loss. Altogether this indicates that plastid-to-nucleus gene transfer has likely been rare in both florideophytes and Bangiales. Cyanidiales contain the next largest plastid gene complements altogether comprising 227 cyanobacterial genes (Figure 2B), suggesting they may too share this characteristic. The stable retention of large plastid gene complements was corroborated by the reconstruction of the ancestral red algal plastid genome: this analysis identified a sum of 261 cyanobacterial genes in red algal plastids (Figure 2B), 43 of which are completely absent in plastids of other algae, where many have been relocated to the nucleus.

### A tRNA Gene Intron Encoding a Maturase Protein

**Figure 3:**
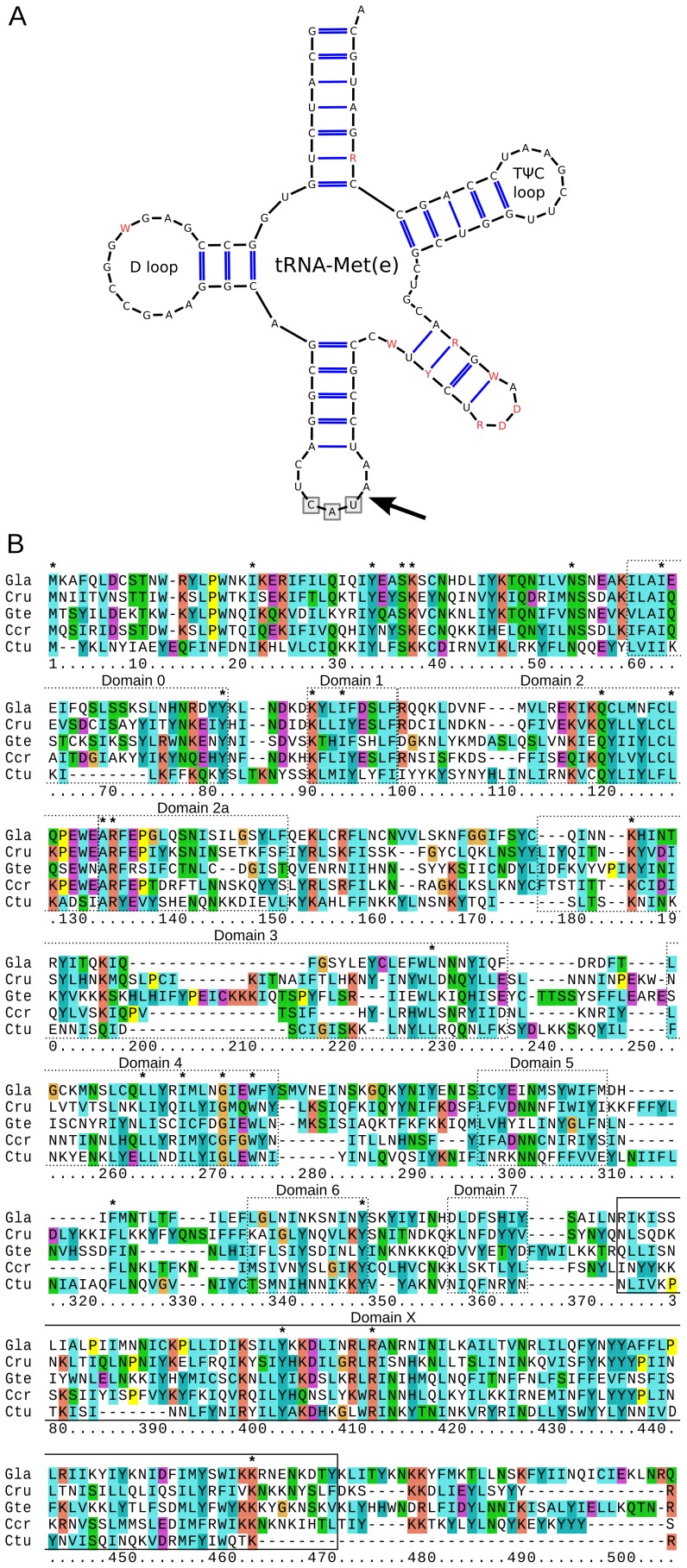
Conserved group II intron in florideophyte plastid *trnMe*. (A) Model of hypothetical secondary structure of the newly identified elongator methionine tRNA showing the position of the intron inserted after the third anticodon position (arrow). Residues in red differ among florideophytes. (B) Sequence alignment of the intron-encoded reverse transcriptase/maturase from five florideophyte plastid genomes with putative reverse transcriptase domains shown by dotted line boxes, the maturase domain shown by a solid line box, and conserved residues shown by asterisks (Gla *Grateloupia lanceola*; Cru *Cruoria* sp.; Gte *Gracilaria tenustipitata*; Ccr *Chondrus crispus*; Ctu *Calliarthron tuberculosum*).

Atypical group II introns have been reported in several plastid protein-coding genes of unicellular red algae [9], [11], [26], [27]. Their secondary structures have not been determined and they do not encode reverse transcriptase/maturase proteins, which are typically involved in intron propagation and maturation. In contrast, none of the already available red algal plastid genomes was reported to contain any introns. However, we noticed that the annotation of the previously published plastid genome of *Gracilaria tenustipitata* var. *liui* lacks an essential gene for elongator tRNA-Met (*trnMe*). Specific searching for this gene revealed it is indeed present, but is difficult to identify because it contains a group II intron. Group II introns in plastid tRNA genes are uncommon and were previously reported only from streptophytes (land plants and charophytes), where they appear evolutionary stable upon acquisition [28], [29]. The predicted *Gracilaria* tRNA-Met folded into a canonical structure with the intron inserted after the third anticodon position (Figure 3A). The intron contains a canonical GTGYG 5′ splice site and conserved domains V and VI. It is also the first red algal intron found to encode a reverse transcriptase/maturase ORF (RT/*mat*). The RT/*mat* contains several putative reverse transcriptase domains and a readily identifiable domain X, which is ubiquitous in RT/*mat*s and required for intron splicing (Figure 3B) [30]. The conserved features of this unusual tRNA/intron pair prompted us to search them in other florideophytes. A homologous RT/*mat*-containing intron in *trnMe* was found in all the four new florideophyte plastid sequences (Figure 3B). Given their large evolutionary distance [12], it is likely that this intron represents a signature characteristic of most florideophytes and hence the majority of red algal diversity. In addition, we found the plastid genome of *Calliarthron* contains a second group II intron in *chlB* displaying conserved 5′ and 3′ motifs and a relatively fast-evolving RT/*mat*. *ChlB* has been lost from the other florideophyte plastids so the gain of this intron cannot be dated, but could have occurred at any point since the time of florideophyte divergence or even earlier, and perhaps concurrently with the *trnMe* intron. The two RT/*mat* proteins were comparatively fast-evolving and their specific affiliations unclear, however, our maximum likelihood phylogenies using a broad sampling of bacterial and plastids homologs showed that both descended from the cyanobacterial and plastid RT/*mat* radiation (data not shown). Interestingly, it has remained present in all five florideophyte species investigated so far, paralleling the stable retention of tRNA gene introns in streptophytes.

### A Unique Horizontal Uptake of a Bacterial Operon into the Plastid Genome

**Figure 4:**
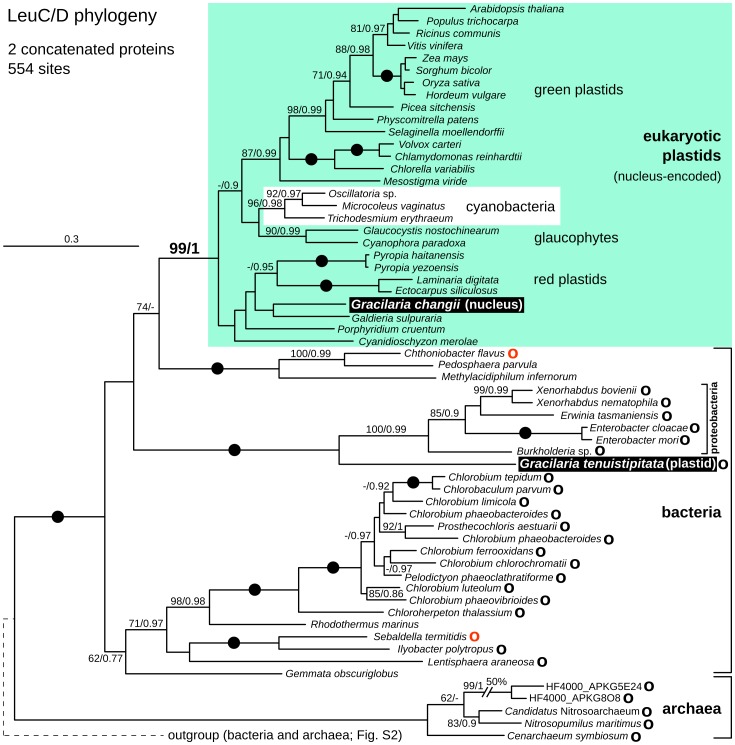
Maximum likelihood phylogeny of concatenated *leuC* and *leuD* genes. Numbers at nodes indicate RAxML rapid boostrap (left) and PhyML SH-aLRT supports (right), and nodes with full support are indicated by black dots. Nucleus-encoded plastid-targeted genes are boxed in green and major lineages labelled to the right. *Gracilaria* sequences are shown in white text on black: *G. changii* has canonical nucleus-encoded *leuC* and *leuD* genes but *G. tenuistipitata* has a plastid-encoded operon that appears to have been derived by recent horizontal gene transfer from a proteobacterial source. Phylogenies of l*euC* and *leuD* are largely congruent, and can be found in Figure S2. Black “O” after species name indicates that *leuC/D* operon is present, red “O” indicates it is intervened by one (*Ch. flavus*) or two short ORFs (*S. termitidis*).

*Gracilaria tenustipitata* var. *liui* plastid genome contains two genes, *leuC* and *leuD*, that are absent in other plastid genomes. The two genes are intact and encode the large and small subunits of 3-isopropylmalate dehydratase (EC 4.2.1.33) catalyzing an isomerization step in leucine biosynthesis and analogous reactions [31]. Leucine biosynthesis also takes place in many other plastids, however, *leuC/D* subunits are always expressed in the cytoplasm and targeted to the plastid post-translationally [32]. The unexpected presence of *leuC* and *leuD* in the *G. tenuistipitata* plastid genome led us examine their phylogeny using a broad sampling of their closest homologs from bacteria, archaea and plastids. Both single-protein phylogenies and a phylogeny based on both proteins concatenated yielded a very similar picture: *G. tenuistipitata leuC* and *leuD* were consistently and strongly related to a clade of six beta- and gamma-proteobacteria, and only distantly related to the clade containing plastid targeted proteins (Figure 4, Figure S2). Interestingly, species representation in both trees was virtually identical: a few topological differences between the two trees appeared at a deeper branching level, however, none of these was significantly supported (Figure S2). This is significant because *leuC* and *leuD* are consistently found adjacent (Figure 4), presumably in a single operon so, while both of the genes have complex phylogenies seemingly indicating multiple horizontal transfer events, they have been inherited and transferred as a single unit still retained in most genomes. Horizontal transfer of protein-coding genes into plastid genomes are exceedingly rare, but often proven important in addressing biochemical or evolutionary questions [33], [34]. This novel case in *G. tenuistipitata* is therefore noteworthy, but also raises interesting questions about its potential function.

Some clue as to this function may be found in a comprehensive search for *leuC/D* variants in all available genomes and transcriptomes from red algae and other photosynthetic eukaryotes. In all cases (except *G. tenuistipitata*), a single type of *leuC* and *leuD* genes was found, and all of these were closely related to the canonical plastid-targeted type found in land plants (Figure S2, Figure 4). Proteins translations of the 5′-complete *leuC* and *leuD* genes contained N-terminal extensions encoding putative transit peptides when examined by TargetP (data not shown) compared to bacterial homologs, altogether indicative of plastid-targeting. Intriguingly, both *leuC* and *leuD* were found in diverse red algae including *leuD* in *Gracilaria changii*, but no significant homologs were detected in the available transcriptomic data from *G. tenuistipitata*. It would appear then, that ancestrally and up to at least one member of the Gracilariaceae family (*G. changii*) red algal plastids use the canonical plastid-targeted *leuC/D*, but that a second proteobacterial-like *leuC/D* was later acquired in *G. tenuistipitata* by horizontal gene transfer into the plastid genome. The plastid *leuC/D* operon is obviously intact and its product is correctly compartmentalized, i.e. potentially functional. If this operon is indeed expressed, then either a canonical plastid-targeted *leuC/D* is co-localized with it, or has already been lost and replaced by the novel plastid-encoded *leuC/D*. To our knowledge, either of these scenarios is unprecedented in organellar evolution.

### Plastid Genome Phylogeny

**Figure 5:**
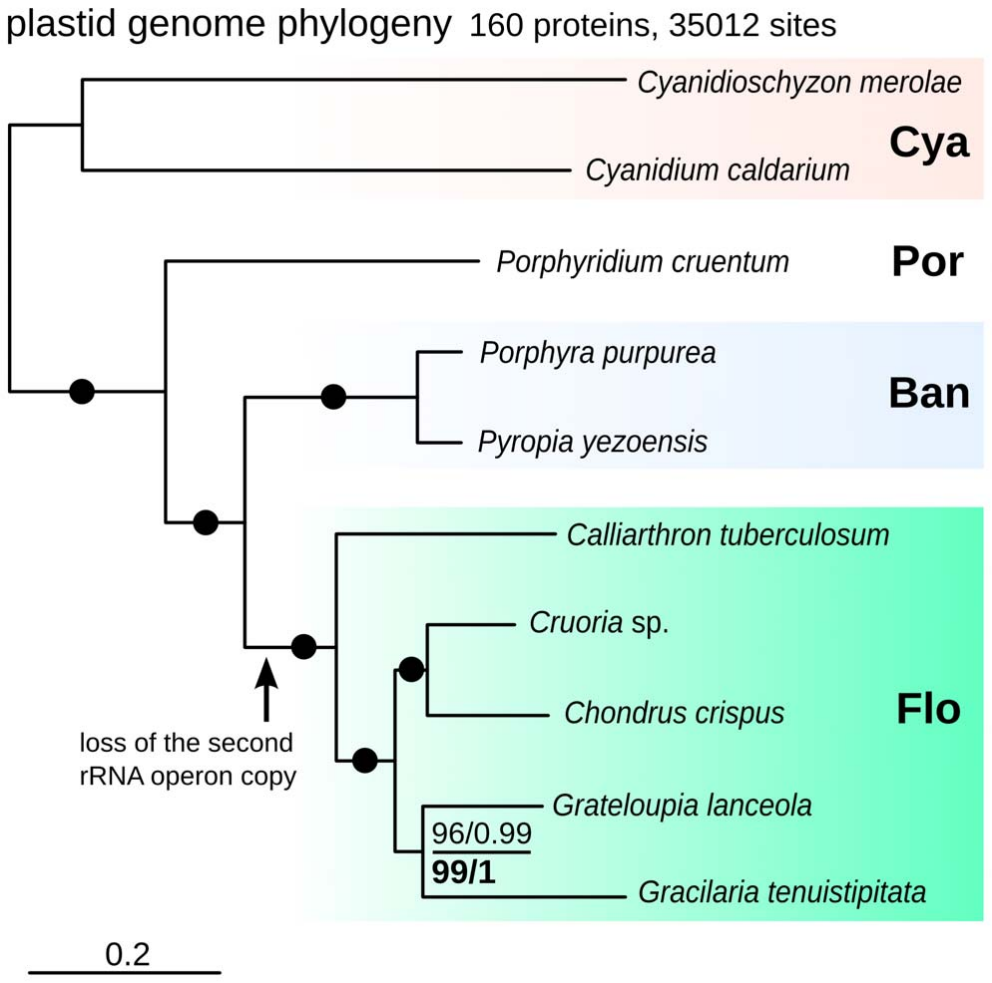
Maximum likelihood phylogeny of 160 concatenated red algal plastid genes. Major lineages are shaded and named to the right (Cya Cyaninidiales; Por Porphyridiales; Ban Bangiales; Flo florideophytes). The phylogeny is fully supported except for the node uniting *G. lanceola* and *G. tenuistipitata*, for which RAxML rapid boostrap (left) and PhyML SH-aLRT supports (right) are shown for trees both including Cyanidiales (above) and excluding them (below).

Multigene phylogenies have already shown great promise in illuminating red algal relationships [11], [12]. Here we inferred the first plastid genome-wide phylogeny using 10 species of red algae. We selected 160 conserved proteins shared across the now eight complete plastid genomes, and extended this dataset by sequences from the partial plastid genome of *Cruoria* sp. and the transcriptome of *Porphyridium cruentum* [7], which contained many plastid-encoded genes. Maximum likelihood (ML) phylogenies of the resulting 35,012 amino acid matrix had complete support at all nodes except the branch uniting *Gracilaria* and *Grateloupia* (96% ML bootstrap and 0.99 SH-aLRT support; Figure 5). When the comparatively fast-evolving Cyanidiales were excluded, the support for the grouping of *Gracilaria* and *Grateloupia* increased (99% ML and 1.0 SH-aLRT support; Figure 5). Assuming the well-established early-branching position of Cyanidiales, the phylogeny revealed an expected sister relationship of the unicellular *Porphyridium* to the monophyletic clade uniting Bangiales and florideophytes. Within florideophytes, *Calliarthron* is early-branching, and *Chondrus* and *Cruoria* were specifically related, consistent with their assignment into different families within a single order, Gigartinales. The phylogenetic affinity between *Gracilaria* and *Grateloupia* points to a possible link between their orders Gracilariales and Halymeniales, whose relationships were previously unresolved [12], and prompts for more extensive plastid genome sampling of other Rhodymeniophycidae orders to further test this. The overall high support for the tree suggests plastid genome-wide phylogenies are a promising way to resolve previously puzzling relationships between florideophyte orders and families, many of which cannot be discriminated easily by morphological data, single gene, or even small multigene phylogenies [12].

### Gene Evolutionary Rates

**Figure 6:**
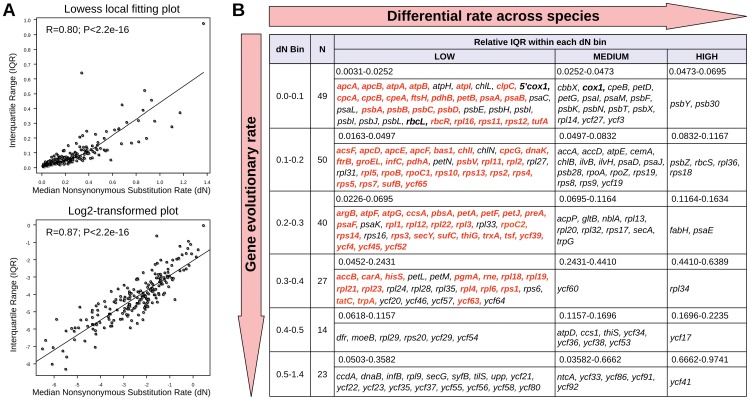
Assessing red algal plastid genes for phylogenetic and barcoding potential. (A) Comparison of median nonsynonymous substitution rate (dN) of protein-coding genes with interquartile range (IQR). The dN is positively correlated with the IQR (lowess local fit scatterplot, top), indicating that genes with higher evolutionary rate tend to show higher rate variations among species. When adjusted for data normality the positive correlation strength increases (log2-transformed scatterplot, bottom). (B) Summary of characteristics of individual genes. genes are binned according to their synonymous rate (vertical axis) and IQR (horizontal axis). Genes with a low IQR (right) and low dN (top) are potentially useful for higher level phylogenetic questions and distant species discrimination, whereas genes with low IQR (right) and medium-to-high dN (mid-to-bottom) are potentially useful for population studies and barcoding at subspecies level. Most promising candidate genes for phylogenies, barcoding, and population studies are in red (see text).

The multicopy nature of plastid genomes makes them an ideal source of easy-to-amplify barcoding markers. Some of these, such as *rbcL*, have already been widely tested, either alone or in combination with other markers such as the 5′ coding region of the mitochondrial *cox1*, and have proven useful for discriminating red algal species [13]–[16], [35]. Molecular markers have also been helpful in unveiling cases where morphology is misleading (e.g., [36]). However, evolution of morphological and molecular traits are not directly correlated, and different taxa may therefore require multiple different markers to achieve a satisfactory resolution in addressing their relationships at different levels (from variation within populations to deep phylogeny). We used the six complete plastid genomes from Bangiales and florideophytes to conduct a pilot comparative study on the evolutionary rates of all red algal plastid genes to see what other genes might be targets for species identification markers. We estimated pairwise values of nonsynonymous substitutions per site (dN) as a proxy for the overall gene divergence rate (substitutions at synonymous sites are saturated and are thus unsuitable for measuring rates of evolution). While this does not test for the presence of a ‘barcode gap’ in a marker, it may indicate which genes are the most plausible candidates for direct testing. We calculated dN for 203 selected plastid genes including the commonly used barcoding markers *rbcL*, the mitochondrial *cox1*, and the 5′coding region of *cox1* (referred to as *5′cox1*) using the maximum likelihood approach. Nonsynonymous substitution rates (dN) and their standard deviation (interquartile range, IQR) differed substantially among the plastid genes (Materials and Methods, Table S2). When median dN estimates were orderly plotted (Figure S3), *rbcL*, *cox1*, and *5′cox1* values were strikingly similar to each other (0.05, 0.06 and 0.04, respectively) and lower than the majority (85–91%) of plastid genes. However, dN was also positively correlated with IQR, suggesting that larger dN is associated with a more unequal dN distances among species, which is an undesirable characteristic for a barcoding or a phylogenetic marker (Figure 6A; R = 0.80–0.87, *P*<2.2e−16). To reflect this variation in both dN and IQR, and highlight the most suitable candidate genes for test barcoding experiments, we sorted all plastid genes into several dN bins and three categories of relative IQR within each dN bin (Figure 6B). This revealed that at least 33 plastid genes are not satisfying candidates for barcoding and phylogenies due to very high nonsynonymous substitution rates (dN >0.5) or highly unequal rates between species (relative IQR = HIGH; see Figure 6). In order to narrow down the list, we limited the most reliable candidates to the lowest IQR category (most equal rates among species), low-to-medium dN rates (dN 0.1–0.4), length over 300 nucleotides, and ubiquitous presence among all taxa. This resulted in 23, 26, 26, and 15 gene candidates with the highest discrimination potential in their respective dN bins (0.0–0.1, 0.1–0.2, 0.2–0.3, 0.3–0.4; genes highlighted in red in Figure 6). Genes in lower dN bins are more likely to provide better resolution in higher level phylogenies and discriminate between distant species. Not surprisingly, the lowest dN bin contains primarily genes for conserved membrane proteins (photosystems, cytochrome *b*_6_ *f*, and ATPase complexes) and other genes that should be sought first when resolving problematic nodes on the red algal tree of life (see [12]). Both of the most commonly used red algal barcoding markers, *rbcL* and *5′ cox1*, also placed in the lowest dN bin showing they have accumulated relatively few nonsynonymous substitution changes among distantly related red algae, and while they have been successfully used in distinguishing many red algal species, they may be less informative at subspecies levels. *RbcL* and 23S rRNA (UPA) have already been shown relatively less sensitive in several florideophyte taxa [15], [16], [37]. Higher dN categories, on the other hand, contain gene candidates with better promise in subspecies and population studies. The majority of plastid genes belong here, some of which may represent good candidates for addressing cryptic species diversity, population structures, and species biogeography, all increasingly studied topics in molecular research on red algae [36], [38], [39]. Indeed, the above division is only approximate and largely dependent on the limited sampling currently available: genes from the medium IQR category may be equally suitable for many barcoding efforts, especially when working with very closely related taxa. For example, *cpeB* previously tested for barcoding callithamnioid red algae [17], and the full length coding sequence of *cox1* fall into the medium IQR category. Similarly, gene length may not be an important factor if a marker is constructed to span a conserved intergenic region (such as the rubisco spacer, [40]); an important aspect for novel marker design considering the highly conserved gene order among Bangiales and florideophytes revealed in the analysis above. Altogether, the gene categories and barcoding marker candidates identified here should be used in guiding phylogenetic dataset assembly and direct ‘barcoding gap’ testing at different taxonomic levels, among which they show a particular promise in sub-species level questions where effective variability of conventional markers, such as *rbcL* or *5′cox1*, may be too low to provide a sufficient taxonomic resolution.

### Conclusions

The three complete and one partial plastid genome described here fill a substantial gap in plastid data representation among florideophytes, the most diverse group of red algae. The data reveals that most red algal plastid genomes have retained highly compacted and gene-rich architectures that are slow-evolving are most closely resemble the ancestral DNA of all plastids. They are largely devoid of unique uknown ORFs, and display highly conserved gene order, with an exception in *Pyropia yezoensis*, where several single gene inversions in canonical gene clusters have occurred. Among their unique features, we describe the first red algal plastid group II intron encoding a reverse transcriptase/maturase in a tRNA gene, and a rare horizontal transfer into the plastid genome of a bacterial operon (*leuC/D*) potentially involved in plastid function. At a more general level, our data demonstrates that plastid genomes are both easily-obtainable using high-throughput shotgun sequencing approaches and promising in resolving red algal relationships, and a genome-wide analysis of gene evolutionary rates identified candidate plastid genes for species barcoding and how they compare to well-established markers, such as *cox1* and *rbcL*.

## Materials and Methods

### DNA Sequencing and Plastid Genome Recovery

The gametophyte of *Chondrus crispus* was collected in Peggy’s Cove, Nova Scotia, Canada (no specific permissions were required for collection of *C. crispus* at this location). The plastid genome was identified on four scaffolds in *Chondrus* nuclear genome project sequence that was assembled using Arachne. The four scaffolds were further assembled with Solexa reads into a single contig (87-fold average coverage) and the joins verified by direct sequencing of PCR-amplified fragments. Collection, DNA extraction, sequencing, and sequence assembly of *Calliarthron tuberculosum* was described previously [7]. A single circularly-mapping plastid genome contig (12-fold average coverage) was identified in the assembled sequence using homology searches. The complete plastid genomes of *Chondrus crispus* and *Calliarthron tuberculosum* were deposited publicly in GenBank (HF562234, KC153978). The plastid genome of *Grateloupia lanceola* was identified on two large contigs (contig00050, HM767098.1, and contig00754, HM767138.1) in 454 sequence assembly of total genomic DNA [6]. The two contigs comprised complementary parts of a complete plastid genome split at two intergenic regions inside conserved gene clusters (*trnF*(gaa)-*clpC* and *apcB*-*atpE*) and contained at their very ends complementary sequence that was part of stem-loop structures conserved among all florideophytes. This clearly indicated that a complete circularly-mapping sequence of *Grateloupia* plastid genome was present in the data that was split at loop regions of two intergenic stem-loop structures suggesting only several bases inside these loop regions are likely to be missing. Several frameshifts at homopolymeric regions in *Calliarthron* and *Grateloupia* plastid genomes introduced by the 454-pyrosequencing approach were corrected manually by comparison to other florideophytes. A partial plastid genome of an undetermined species from the Cruoriaceae family was recovered on ten contigs 454 sequence contigs of total genomic DNA [6]. A single 18S ribosomal RNA sequence 99% similar to *Cruoria pellita* was recovered from the assembly (contig02333, HM765770.1) leading us to refer to this species as *Cruoria* sp. Altogether, 10 plastid contigs totalling 106493 base pairs of *Cruoria* sp. were identified (accessions HM765655.1, HM765656.1, HM765658.1, HM765667.1, HM765668.1, HM765669.1, HM765678.1, HM765680.1, HM765788.1, HM765812.1).

### Annotations, Genome Comparison and Intron Analysis

All plastid genomes were annotated manually using Artemis 13.2 [41]. Publicly available red algal plastid genomes (GenBank accessions are listed in Table 1) were re-annotated in accordance with the new sequences and changes ORF predictions were listed in Table S1. The plastid genome of *Porphyra umbilicalis* had an identical gene content and order to that of closely related *P. purpurea* and was excluded from further analysis. Open reading frames longer than 100 amino acids were annotated. Ten protein frameshifts were detected in the published plastid genomes and corrected by using ambiguous nucleotides (Table S1). Start codons of protein-coding genes predicted according conservation of N-terminal regions across red algal and red alga-derived plastids. Ribosomal RNA, tRNA and small RNA genes were predicted using RNAmmer 1.2 Server [42], tRNAscan-SE 1.21 [43] and ARAGORN [44], and Bcheck rnpB Server [45], respectively. All updated annotations are available upon request. Group II intron boundaries were predicted according to 5′ and 3′ conserved motifs, and integrity of1 gene sequences. Domains V and VI were identified based on their conserved secondary structure, while domains I-IV could not be readily folded. Approximate domain boundaries in intronic reverse transcriptases were determined by aligning them to the dataset available at Mobile group II intron database [46] (http://www.fp.ucalgary.ca/group2introns/) and visualized using ClustalX 2.1 (Figure 3B). Genomes were cross-compared using Artemis Comparison Tool 10.2 [47] (Figure S1) and MAUVE 2.3.1 [48] (Figure 1). MAUVE was run under the Progressive Mauve algorithm using ‘Use seed families’ option and arbitrary Match Seed Weight = 17. Cyanobacterial gene content (Figure 2B) was visualized with the help of the R package VennDiagram [49]. Rubisco, *menA*-*F*, and *leuC/D* genes, and uknown ORFs were exluded from the cyanobacterial gene count. Figures were drawn and manipulated using Inkscape vector graphic editor (0.48) and GIMP image editor.

### Phylogenetic Analyses and Gene Evolutionary Rates

*LeuC* and *leuD* sequences were extracted from GenBank protein and EST databases using closest homologs from blastp homology searches. Plastid genome dataset was assembled using custom scripts. Protein datasets were aligned using ‘–localpair’ algoritm in MAFFT 6.857b [50], and the alignment trimmed using Gblocks 0.91b [51] using ‘b1 = 50%+1, b2 = 50%+1, b3 = 12, b4 = 4, b5 = h’ parameters. The final plastid genome matrix contained almost full data representation (0–1% of missing data) from all species, except *Porphyridium cruentum* (21%) and *Cruoria* sp. (45%). Phylogenetic inferences were done in RAxML 7.28 [52] using ‘-m PROTGAMMALGF -f a -# 1000’ parameters and PhyML 3.0.1 [53] using ‘-m LG -f e -v e -c 8 -a e -s BEST -b -4 -n_rand_starts 10’ parameters. Trees were drawn with the help of FigTree. Prior to the determination of gene evolutionary rates, nucleotide sequences of 203 selected Bangiales and florideophyte plastid genes (i.e., all protein-coding genes present in at least three different taxa; uknown ORFs were excluded) were aligned based on their amino acid translations using MUSCLE [54] using the default settings and an in-house Perl script (available upon request). The aligned nucleotide sequence matrix was manually checked using BioEdit [55] and pairwise nonsynonymous substitution rates (dN) estimated using the maximum likelihood approach [56]. The ‘mold, protozoan, and coelenterate’ mitochondrial genetic code was used for the dN estimation of *cox1*, whereas the standard genetic code was used for plastid genes. A total of 2956 pairwise dN comparisons were calculated among the 203 genes by using Codeml implemented in PAML [56] with the aid of an in-house Perl script (available upon request). Pairwise dN estimates for each gene were summarized in boxplots (Figure S3) using the function “boxplot” in the statistical package R (www.r-project.org). The Pearson’s product-moment correlation analysis was implemented to examine the association between median dN and interquartile range (IQR) using R.

## Supporting Information

Figure S1 Single gene inversions in *Pyropia*. Linearized maps of plastid ribosomal super cluster from the Bangiales *Porphyra* (top) and *Pyropia* (bottom). *Pyropia* has three single gene inversions relative to *Porphyra*, and all other red algal and secondary red alga-derived plastid genomes.
(TIFF)

Figure S2 Maximum likelihood phylogenies of *leuC* and *leuD* individually. numbers at nodes correspond to RAxML rapid boostrap (left) and PhyML SH-aLRT supports (right). The topologies are broadly consistent with the concatenated phylogeny shown in Figure 4.
(TIFF)

Figure S3 Plastid gene dN rates. Boxplot is showing relative nonsynonymous substitution rates (dN) and their standard deviation (interquartile range; IQR) for all selected red algal plastid genes including *rbcL*, mitochondrial c*ox1* and 5′ coding region of cox1 (*5*′*cox1*). Median is indicated by a solid line, the sample minimum and maximum are indicated by dotted lines and bars outside the box, and outliers are indicated by open circles. For comparison, the positions of *rbcL* and a dN of 0.5 are highlighted.
(TIFF)

Table S1 Annotation changes in publicly available red algal plastid genomes.
(XLS)

Table S2 Boxplot statistics of nonsynonymous substitution rates estimated using Maximum likelihood.
(XLS)
