# Supplementary figures and images for: Evolution of Red Algal Plastid Genomes: Ancient Architectures, Introns, Horizontal Gene Transfer, and Taxonomic Utility of Plastid Markers
Source: PLoS One. 2013 Mar 25;8(3):e59001. doi: 10.1371/journal.pone.0059001 (PMC3607583; doi:10.1371/journal.pone.0059001)

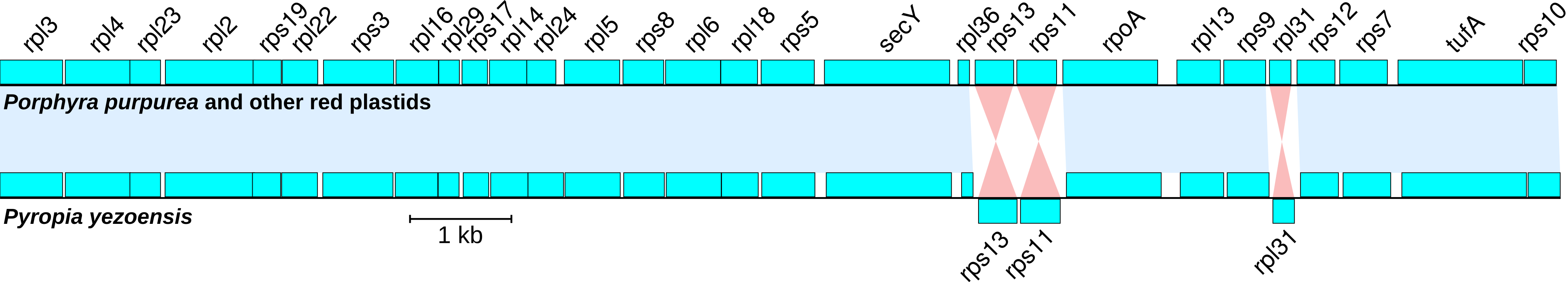

Supplement: Figure S1 — Single gene inversions in Pyropia. Linearized maps of plastid ribosomal super cluster from the Bangiales Porphyra (top) and Pyropia (bottom). Pyropia has three single gene inversions relative to Porphyra, and all other red algal and secondary red alga-derived plastid genomes. (TIFF) [file pone.0059001.s001.tiff]

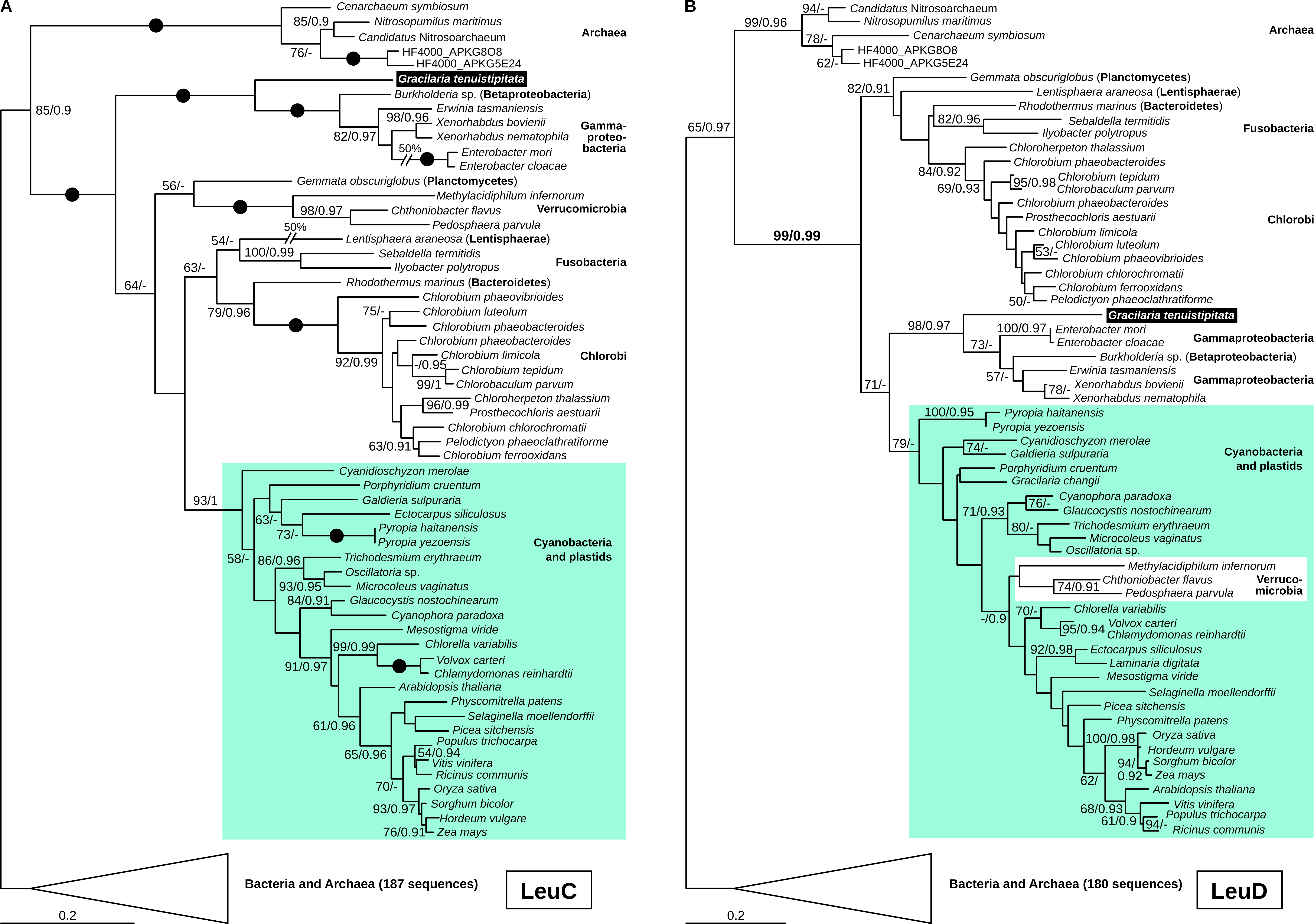

Supplement: Figure S2 — Maximum likelihood phylogenies of leuC and leuD individually. numbers at nodes correspond to RAxML rapid boostrap (left) and PhyML SH-aLRT supports (right). The topologies are broadly consistent with the concatenated phylogeny shown in Figure 4. (TIFF) [file pone.0059001.s002.tiff]

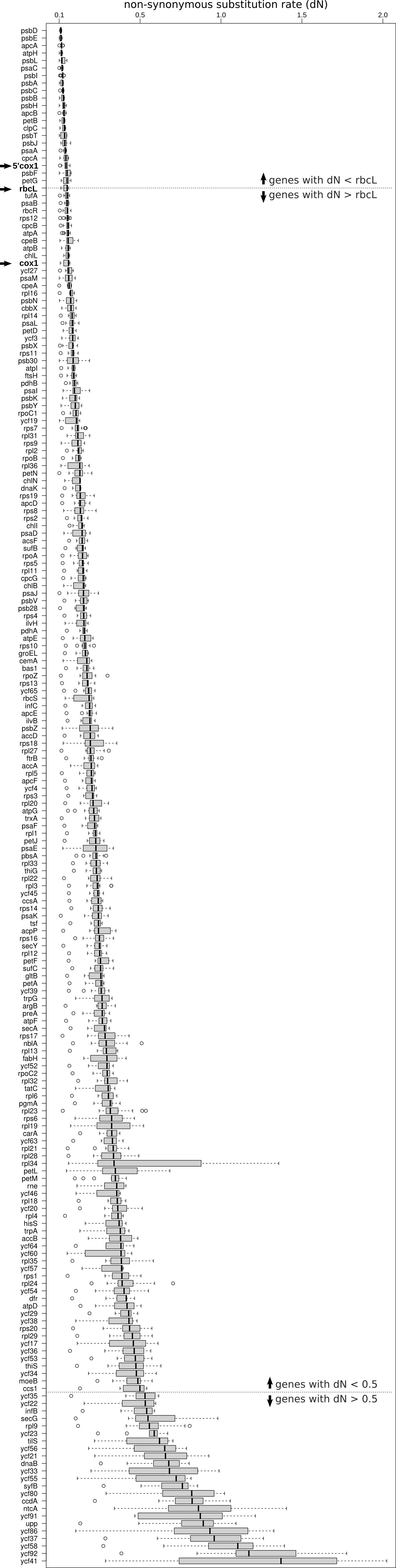

Supplement: Figure S3 — Plastid gene dN rates. Boxplot is showing relative nonsynonymous substitution rates (dN) and their standard deviation (interquartile range; IQR) for all selected red algal plastid genes including rbcL, mitochondrial cox1 and 5′ coding region of cox1 (5′cox1). Median is indicated by a solid line, the sample minimum and maximum are indicated by dotted lines and bars outside the box, and outliers are indicated by open circles. For comparison, the positions of rbcL and a dN of 0.5 are highlighted. (TIFF) [file pone.0059001.s003.tiff]
